# β-endorphin modulates the effect of stress on novelty-suppressed feeding

**DOI:** 10.3389/fnbeh.2013.00019

**Published:** 2013-03-14

**Authors:** Elizabeth T. Barfield, V. Alexandra Moser, Annie Hand, Judith E. Grisel

**Affiliations:** ^1^Department of Neuroscience, Furman UniversityGreenville, SC, USA; ^2^Neuroscience Graduate Program, University of Southern CaliforniaLos Angeles, CA, USA; ^3^Department of Psychology, Bucknell UniversityLewisburg, PA, USA

**Keywords:** opioids, transgenic, anxiety, depression, mice, hyponeophagia, novelty

## Abstract

Although stress is implicated in the pathophysiology of mood and anxiety disorders, not all individuals who suffer stressful life events develop psychopathology. Differential susceptibility to stress may be influenced by genetically mediated differences in hypothalamic-pituitary-adrenal (HPA) axis activity and moderation of the stress response by the opioid peptide β-endorphin (β-E). The present study investigated genetic contributions to coping behavior by examining anxious behavior of transgenic mice with varying capacities to synthesize β-E [B6.129S2-*Pomc*^*tm1Low*^/J; regulated by insertion of a premature stop codon into one or both copies of the proopiomelanocortin (*POMC*) gene], both under normal conditions and following 3 min of forced swim (FS). Ten minutes after this stress exposure or a control manipulation, acutely food-deprived female and male transgenic mice were subjected to a novelty-suppressed feeding (NSF) test, during which their interaction with an almond slice located in the center of an open field box was measured. There was an interaction between genotype and stress for latency to approach the almond and whether or not the almond was approached, such that mice with low or absent β-E displayed a stronger aversion to novelty-feeding after stress exposure than did mice with normal levels. These data provide evidence for a moderating effect of β-E on the behavioral response to stress. Genotypic differences in anxious behavior emerged when mice were stressed prior to behavioral assessment, suggesting that β-E plays a role in coping behavior. These findings indicate that genetic variability in sensitivity of the β-E system to stress may contribute, at least in part, to heritable differences in stress reactivity as well as vulnerability to stress-related psychopathology.

## Introduction

Over the past few decades, an extensive body of work has emerged linking vulnerability to affective and anxiety disorders with stressful life events. Stressful events often precipitate depressive episodes (Brown et al., 1987; Hammen et al., 1992), and early life stress has been shown to increase the risk for stress-related psychiatric disorders in adulthood (Kendler et al., 1992a; McEwen, 2003). However, not all individuals who suffer stressful life events develop psychopathology; evidence suggests that some individuals are resistant, and others vulnerable, to the adverse effects of stress (de Rijk and de Kloet, 2005; Southwick et al., 2005; Stiller et al., 2011; Castro et al., 2012; see Sandi and Richter-Levin, 2009, for review). Differential vulnerability to stress is regulated by an interaction of genetic and developmental factors with major life stressors (Sullivan et al., 2000; Danese, 2008; Bet et al., 2009). However, the neurobiological mechanisms underlying susceptibility to stress-related disorders remains poorly understood.

One hypothesis is that genetic factors influence coping style to moderate the vulnerability to stress (see Feder et al., 2009, for review). “Coping” describes the behavioral and physiological mechanisms that occur to return an organism to a basal state following stress exposure. Thus, less effective coping, defined as a failure to recover to a baseline state after stress exposure, may render an individual more susceptible to stress-induced psychopathology (McEwen, 2002; Meng et al., 2011). For example, in rats, a behavioral profile characterized by high anxiety is associated with susceptibility to the development of stress-induced depression-like behavior (Sandi et al., 2008; Stedenfeld et al., 2011; Castro et al., 2012). In humans, the neuroticism-anxiety trait, which is associated with disengagement coping (an ineffective strategy; see Carver and Connor-Smith, 2010 for a review of personality and coping) and less flexible coping strategies across situations (Lee-Bagley et al., 2005), strongly reflects liability to major depressive disorder (MDD) and generalized anxiety disorder (GAD; Kendler et al., 2006a, 2007).

Moreover, differences in coping behavior and vulnerability to stress may have a biological basis in hypothalamic-pituitary-adrenal (HPA) axis function (van Santen et al., 2011) and the moderating effects of the endogenous opioid peptide β-endorphin (β-E) on the stress response (Schedlowski et al., 1995; Gianoulakis, 1998; Sarkar et al., 2007; Grisel et al., 2008; Barfield et al., 2010). Activation of the HPA axis following exposure to stressful stimuli mediates an adaptive response through a hormonal cascade of behavioral and physiological changes aimed at the maintenance of homeostasis in the body (Low, 2004). During stress, the secretion of corticotrophin releasing hormone (CRH) stimulates expression of the proopiomelanocortin (*POMC*) gene in the anterior pituitary, which is subsequently translated into peptides such as adrenocorticotropic hormone (ACTH) and β-E (Charmandari et al., 2005). While ACTH activates the adrenal gland to initiate the peripheral response to stress, β-E attenuates the stress response, at least in part, by inhibiting secretion of CRH (Buckingham, 1986; Plotsky, 1991) and blocking stress-induced nociception (Bodnar et al., 1980; Nakagawasai et al., 1999; Parikh et al., 2011). Reports indicating modulation of the HPA axis by β-E fit well with those evincing a role for β-E in the behavioral response to stress (Amir, 1982; Yamada and Nabeshima, 1995; Ribeiro et al., 2005; Grisel et al., 2008; Barfield et al., 2010). For example, we have shown that transgenic mice with low β-E exhibit increased anxious behavior and show deficits in coping ability during an inescapable aversive situation (Grisel et al., 2008; Barfield et al., 2010). Thus, because stress-induced release of β-E mediates endocrine and behavioral responses that contribute to allostasis of the stress response, insufficient attenuation of the HPA axis arising from low β-E may contribute to maladaptive coping behavior under stressful conditions.

Here, we examined the role of β-E in anxious behavior of mice, both under basal conditions and following exposure to an acute stressor. Anxious behavior was assessed using the novelty-suppressed feeding (NSF) test, an ethologically relevant paradigm that measures the suppression of food intake (in a food-deprived animal) caused by exposure to a potentially anxiogenic novel environment (typically an open field; Merali et al., 2003; see Cryan and Sweeney, 2011 for summary of hyponeophagia paradigms). Because anxiolytics and chronic but not acute antidepressants reduce hyponeophagia (Britton and Britton, 1981; Shephard et al., 1985; Bodnoff et al., 1988; Bessa et al., 2009), the NSF test provides a sensitive and reliable measure of anxiety-related states in animals that resemble those in humans (Merali et al., 2003). Thus, we assessed the effect of genotype (β-E level) and previous stress exposure, as well as their interaction, on anxious behavior in the NSF test. We hypothesized that studying the behavioral response to stress in mice with varying levels of β-E would reveal an interaction of genetic predisposition and environmental stress, such that differences in coping behavior between genotypes would emerge following stress exposure.

## Materials and methods

### Subjects and design

Subjects were adult naïve male and female wild-type (C57BL/6J; B6), heterozygous (HT), and β-E-deficient (B6.129S2-*Pomc*^*tm1Low*^/J; KO) mice. Transgenic mice were developed over a decade ago in the laboratory of Malcolm Low (Rubinstein et al., 1996) by insertion of a premature stop codon into the *Pomc* gene. Homozygotes (KO) are entirely unable to synthesize β-E, though all other *Pomc* products show normal expression. Opioid receptor expression also remains unchanged (Rubinstein et al., 1996). Mice for these studies were bred in-house from stock purchased from Jackson Laboratories (Bar Harbor, ME, USA). The gene mutation has been fully backcrossed to the C57BL/6J strain (>20 generations). HT mice were bred from KO males and B6 females; others were bred under identical conditions from genotype-matched pairs. Mice were weaned at 21 days of age and were group-housed by sex with 3–4 per Plexiglas cage, measuring 20 × 35 × 14.5 cm. Mice were maintained in a colony room at 21 ± 2°C, on a reverse 12:12 light:dark cycle with lights on at 7 p.m. Water and food were available *ad libitum*. All procedures were carried out in accordance with the National Institutes of Health guidelines and approved by the Animal Care and Use Committee of Furman University.

### Behavioral testing

On testing day, food was removed at ~8 a.m., 1 h after lights-out, in order to facilitate feeding (LeSauter et al., 2009). Behavioral testing occurred during the animals' active phase, between 10 a.m. and 4 p.m., in a dimly lit testing room, so as to enable behavioral assessment of genotypic differences (Branchi and Ricceri, 2002; Hossain et al., 2004; Roedel et al., 2006). Mice were brought into the testing area, weighed, tail marked, and randomly assigned to the control or the forced swim (FS) condition.

Mice in the control condition were individually placed in a Plexiglas cage in the testing room for a 10 min habituation period. Mice in the FS condition were subject to a modified version of Porsolt et al.'s (1977) FS Test for 3 min in a white plastic 5 gallon bucket measuring 30 cm in diameter by 40 cm in height containing 20 cm of water maintained at 23°C. To minimize the possibility of confounding effects (e.g., fatigue) from sex and genotypic differences in behavior (previously reported for 15 min of FS exposure, Barfield et al., 2010), pilot testing was conducted to determine a FS duration that would induce subthreshold amounts of stress. Duration of 3 min was chosen because no sex or genotypic differences in immobile behavior emerged following this brief length of time. Mice were judged immobile when making no movements other than those required to stay afloat, for at least 5 s. Two independent observers recorded latency to immobility, total time spent immobile, and number of immobile segments. Following the FS, mice were individually placed in a Plexiglas cage in the testing room for a 7 min habituation period.

After habituation in the testing room, mice from both the control and the FS conditions were subject to the NSF test (Britton and Britton, 1981; Bodnoff et al., 1988). Mice were individually placed in an open field box (100 × 100 × 4.5 cm) that contained a pre-weighed almond slice in the center, for 5 min. Two independent observers recorded whether the almond was approached, the latency to approach the almond, and the number of times that the mouse sniffed the almond. Following the NSF test, the almond slice was weighed, and the amount of almond eaten was recorded.

### Statistical analysis

Main effects of and interactions between genotype (B6, HT, KO), sex, and stress condition (control, FST) were analyzed using between-subjects analysis of variance (ANOVA). Significant main effects and interactions were further examined using Fisher's least significant difference (LSD) test. Three separate Two-Way chi-square tests of independence were performed to determine whether correlations existed between (1) approach behavior (whether or not the almond was approached) and stress condition (2 × 2 design), (2) approach behavior and genotype (2 × 3 design), and (3) approach behavior and genotype with condition (2 × 6 design). Statistical analyses were performed using SPSS Statistics 17.0 (SPSS, Inc., Chicago, IL). In all cases, the criterion for significance (α level) was set at *p* ≤ 0.05.

## Results

There were no main effects of or interactions with sex, so male and female data were collapsed for all analyses. As expected, there were no main effects of genotype on any measure of immobility in the 3 min FS exposure.

**Figure 1:**
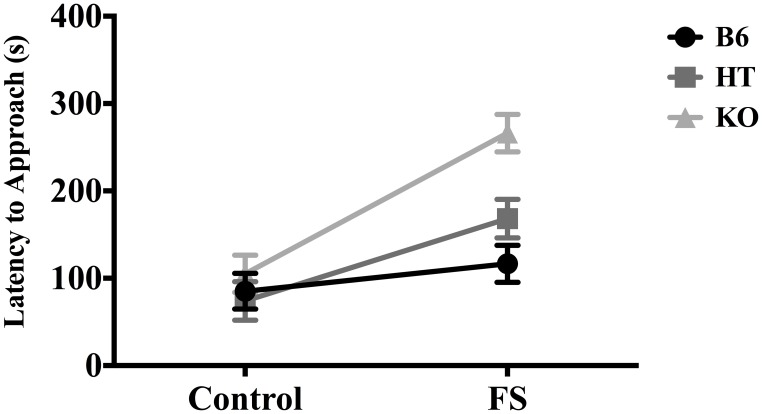
**Latency to approach almond slice during the 5-min novelty-suppressed feeding (NSF) test in wild-type C57BL/6J (B6), heterozygous (HT), and β-E knock-out (KO) mice, following either 10 min single housing (Control) or 3 min of forced swim, followed by 7 min single housing (FS).** Data show mean ± SE. Significant differences (*p*-values ≤ 0.05) between groups were determined following ANOVA by *post-hoc* analysis (Fisher's LSD test). There were main effects of the FS stressor, genotype, and an interaction between stress condition and genotype.

In terms of latency to approach the almond, there was a main effect of condition [*F*_(1, 81)_ = 31.261, *p* < 0.001] and a main effect of genotype [*F*_(2, 80)_ = 9.696, *p* < 0.001]. *Post-hoc* analysis (Fisher's LSD) indicated that KOs took the longest to approach the almond (*p* < 0.01) and differed from both B6s and HTs, which did not differ from each other. There was also a significant interaction between genotype and condition for latency to approach the almond [*F*_(2, 80)_ = 4.899, *p* ≤ 0.01], such that the effect of stress on hyponeophagia increased as β-E levels decreased (Figure 1). There were no genotypic differences in the control condition, but in the stressed condition, KOs took the longest to approach the almond (as confirmed by Fisher's LSD, *p* < 0.05). Thus, the main effect of genotype on latency to approach the almond was driven primarily by differences between genotypes in the stressed condition.

**Figure 2:**
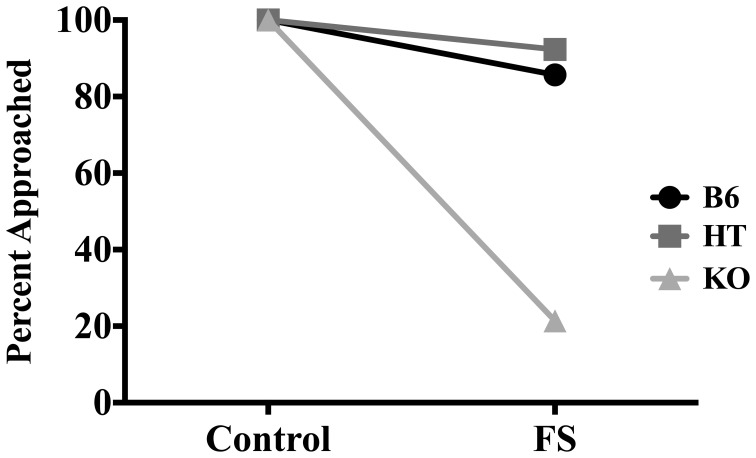
**Percentage of wild-type C57BL/6J (B6), heterozygous (HT), and β-E knock-out (KO) mice in each group that approached the almond slice at least once during the 5-min NSF test.** Significant correlations between experimental variables were determined by Two-Way chi-square tests of independence. Whether or not the almond was approached depended on stress condition, genotype, and interaction of genotype and stress condition (all *p*-values ≤ 0.05).

In terms of whether or not the almond was approached, FS exposure decreased the likelihood that mice would approach the almond at least once during the 5-min NSF test [*X*^2^ (1, *N* = 83) = 17.250, *p* < 0.01]. When data were collapsed across condition, whether the almond was approached depended on genotype [*X*^2^ (2, *N* = 83) = 15.235, *p* < 0.01] such that as β-E levels decreased, likelihood of approaching the almond also decreased. To determine if genotype and condition were correlated with approach behavior, we further separated genotypes into groups based on condition (i.e., B6 stress, B6 control, HT stress, etc.). Whether or not the almond was approached depended on both genotype and condition [*X*^2^ (5, *N* = 83) = 49.427, *p* < 0.01]. Figure 2 depicts the percentages of mice in each genotype and condition that approached the almond. All control mice approached the almond, but whether or not stressed mice approached depended on genotype. Thus, the significant correlation between genotype (collapsed across condition) and whether the almond was approached was driven by genotypic differences that emerged only in the stressed condition.

**Figure 3:**
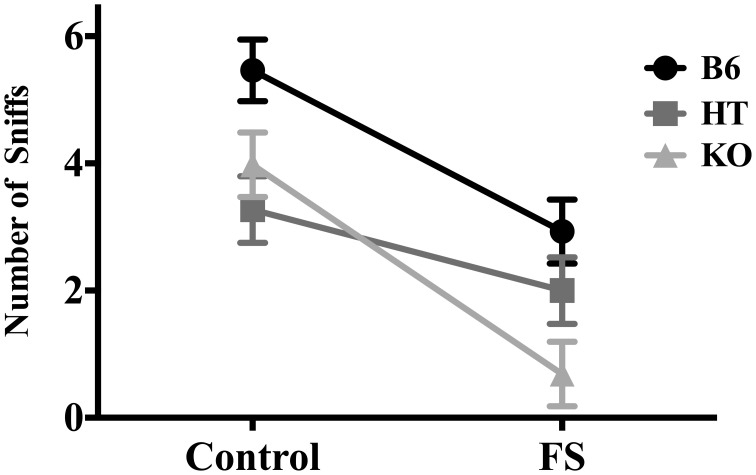
**Number of sniffs to the almond slice made by wild-type C57BL/6J (B6), heterozygous (HT), and β-E knock-out (KO) mice during the 5-min NSF test.** Data show mean ± SE. ANOVA and *post-hoc* analysis (Fisher's LSD test) indicated that forced swim exposure decreased almond sniffing, and a main effect of genotype reflected the fact that B6s sniffed the almond more than either of the other two lines, which did not differ from each other (*p*-values ≤ 0.05). There were no significant interaction effects of genotype and condition on number of sniffs.

For number of sniffs, there was a main effect of condition [*F*_(1, 81)_ = 31.261, *p* < 0.001] such that stressed mice sniffed the almond less frequently, and a main effect of genotype [*F*_(2, 80)_ = 8.681, *p* < 0.001] such that β-E levels were indirectly correlated with degree of hyponeophagia (Figure 3). *Post-hoc* analysis indicated that B6s sniffed the almond more than either of the other two lines (*p* ≤ 0.001), but HTs and KOs did not differ from each other. There were no significant interaction effects of genotype and condition on number of sniffs [*F*_(2, 80)_ = 2.043, *p* > 0.05].

**Figure 4:**
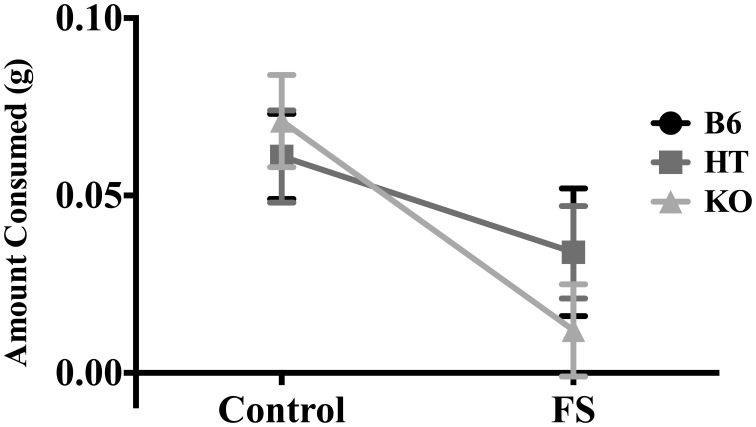
**Average amount of almond eaten during the 5-min NSF test by wild-type C57BL/6J (B6), heterozygous (HT), and β-E knock-out (KO) mice.** Data show mean ± SE. ANOVA indicated that prior forced swim decreased consumption (*p* ≤ 0.05), but there were no differences between genotypes, nor was this effect dependent upon genotype.

There was a main effect of condition on amount of almond eaten [*F*_(1, 81)_ = 17.470, *p* < 0.001] such that stressed mice ate less (Figure 4). However, there was no main effect of genotype [*F*_(2, 80)_ = 0.107, *p* > 0.05] nor an interaction between genotype and condition for this measure [*F*_(2, 80)_ = 0.727, *p* > 0.05].

## Discussion

Employing the NSF test to assess anxious behavior in transgenic mice expressing varying levels of β-E, our findings suggest that β-E modulates the effect of stress on behavior. The ability of exposure to a novel environment to suppress interaction with and ingestion of a highly palatable food was magnified when mice were first exposed to FS in a genotype-dependent manner. (Figures 1, 2). These data are in line with previous reports showing increased hyponeophagia in rodents exposed to unpredictable chronic mild stress (Bessa et al., 2009) or social isolation (Voikar et al., 2005), and extend these findings by suggesting a critical role for β-E.

The main effect of genotype on number of sniffs (Figure 3) suggests a direct relationship between peptide levels and interaction with the novel food stimulus. However, there was also an interaction between genotype and stress for latency to approach the almond (Figure 1) and whether the almond was approached (Figure 2); mice with lower levels of β-E displayed a stronger aversion to novelty-feeding after exposure to stress than did mice with higher levels. Moreover, there were no differences between wild-type (B6), HT, or, β-E knock-out (KO) mice under control conditions. Because the effects of stress on hyponeophagia are magnified with lower levels of β-E, these data suggest that β-E plays an active role in coping behavior by mitigating the behavioral response to stress.

As expected based on pilot testing, there were no effects of or interactions between genotype and sex on immobile behavior of mice during the 3 min FS. In a previous study in our lab, using the same three strains of mice, we found effects of sex and genotype on immobility during a 15 min FS Test (Barfield et al., 2010). However, for the present study, we aimed to induce a subthreshold amount of stress that would not produce genotypic or sex differences in behavior during the FS so as to minimize the possibility that behavior in the NSF test would be confounded by factors such as fatigue from the FS. We found that 3 min of exposure to the FST was just stressful enough for genotypic differences in novelty-feeding to emerge. Furthermore, although we found an interaction between genotype and stress for latency to approach the almond and whether or not the almond was approached, there was no such interaction for number of sniffs and amount of almond eaten. It is possible that interactions between genotype and stress for the latter two measures may emerge with FS times longer than 3 min.

Likewise, although we found no effects of sex on behavior during the NSF test, it is possible that this design did not induce sufficient stressor intensity to allow for detection of sex differences. Thus, the present findings do not preclude the possibility of sex differences in coping behavior. Given that sex differences in the risk for and prevalence of stress-related disorders in humans are well-documented (Kessler et al., 1993; Zilberman et al., 2003; Marcus et al., 2005; Hasin et al., 2007), future research should aim to develop animal models that reflect such differences.

The findings presented here support our earlier findings using the plus maze, light-dark box (Grisel et al., 2008), FS Test, and tail suspension test (TST; Barfield et al., 2010), suggesting that β-E contributes to the ability to behaviorally manage stressful stimuli. For example, we have shown an inverse relationship between β-E levels and anxious behavior (as measured by percent of open arm entries and time spent in the open arms in the plus maze, and time spent in the light compartment of the light-dark box; Grisel et al., 2008). We have also shown a direct relationship between β-E levels and immobility in the FST and TST (Barfield et al., 2010). Because these tests subject mice to inescapable aversive situations, whereby failure to exhibit actions aimed at escape may represent an effective coping strategy, these results suggest that β-E facilitates coping behavior. The present study provides additional evidence to support this role of β-E by showing that under conditions of acute stress, mice alter their behavior in an anxiogenic situation to mitigate a deficiency in β-E. Moreover, these data extend our earlier findings to suggest that behavior becomes increasingly influenced by underlying neurobiology when an organism is exposed to stressors.

An interaction of both genetic predisposition and environmental stressors contributes to increased risk for developing stress-induced psychopathology (Danese, 2008; Bet et al., 2009). In line with this view, it is possible that an individual who produces lower than normal amounts of β-E may suffer from an overactive HPA axis and an impaired ability to effectively manage stressful stimuli (behaviorally and physiologically). These factors may render an individual particularly susceptible to the aversive effects of stress and to developing anxiety and depression. Indeed, evidence from studies utilizing selectively bred rodent lines suggests that individual differences in HPA activity and anxiety traits may contribute to differential susceptibility to stress. Rats with a behavioral profile characterized by high anxiety and low exploration are particularly vulnerable to developing depression-like behaviors and HPA axis hyper-reactivity when exposed to subchronic stress, while low anxiety rats are more resistant to the development of stress-induced depression-like behavior (Castro et al., 2012). Additionally, rats classified as low-responders to novelty (high anxiety) who are exposed to chronic mild stress exhibit increased latencies to approach and consume food in the NSF test, while the behavior of rats classified as high-responders to novelty (low anxiety) is unaffected by chronic stress (Stedenfeld et al., 2011). Chronically stressed low-responder rats also become anhedonic more rapidly and to a greater degree than chronically stressed high-responder rats (Stedenfeld et al., 2011).

The role of genetic factors in the etiology of MDD and anxiety disorders is well recognized (Unschuld et al., 2009; see Sullivan et al., 2000, for review), as heritability is estimated to be around 40% for MDD (Kendler et al., 2006b), and 32% for GAD; Hettema et al., 2001. At least in part because of the complexity of these disorders, candidate gene studies have not been able to unambiguously identify susceptibility genes (Levinson, 2006). Moreover, the high comorbidity of MDD and anxiety disorders (Gorman, 1996; Kessler et al., 1996, 2008; Kaufman and Charney, 2000; Hettema et al., 2003) suggests that risk factors for these disorders are not mutually exclusive (Krueger, 1999; Ohara et al., 1999; Vollebergh et al., 2001; Gorwood, 2004). Indeed, twin studies indicate significant overlap of genetic risk factors for depression and anxiety (Hettema et al., 2003; Kendler et al., 2007). In particular, it has been suggested that the genes influencing liability to MDD are the same as those influencing liability to GAD (Kendler et al., 1992b). Nevertheless, genome-wide association studies suggest that a large number of genes, each with a small effect, influence susceptibility to MDD, and there is overlap in genetic risk factors with GAD (Demirkan et al., 2011).

Because the ability to cope with stress is an important factor influencing susceptibility to stress-related disorders (Meng et al., 2011; Mahmoud et al., 2012), it is possible that shared liability genes for anxiety and depression influence stress reactivity (Kendler et al., 1991; Gorwood, 2004; Yu et al., 2012). Indeed, stress-induced activation of the HPA axis is moderately to highly heritable (Federenko et al., 2004). Healthy individuals with depressed first-degree relatives show a moderately elevated cortisol response following challenge with dexamethasone (DEX-CRH test), though not as elevated as that of patients with MDD (Holsboer et al., 1995), and healthy individuals with diagnosed parental history of anxiety or depression show higher cortisol awakening levels than individuals without parental history (Vreeburg et al., 2010). Moreover, the response of the β-E system to acute stress exposure is also highly heritable (Dai et al., 2002, 2005), and genetic variation in the μ-opioid receptor contributes to the differential response of the HPA axis to stress (Chong et al., 2006; Schwandt et al., 2011).

Although a compelling number of studies report evidence for dysregulation of the HPA axis in patients suffering from depression and anxiety (Young et al., 1991; Carroll et al., 2007; Lloyd and Nemeroff, 2011), the above findings suggest that a hyperactive HPA axis in normal individuals may represent a vulnerability marker for stress-related psychopathology. Because β-E plays a role in moderating the effects of stress (Amir, 1982; Yamada and Nabeshima, 1995) as well as termination of the stress response (Buckingham, 1986), individual differences in HPA axis activation and subsequent release of β-E may influence differential vulnerability to stress-induced changes in the coordination and dynamics of the stress response. Indeed, depressed patients show hypertrophy of the adrenal gland (Rubin et al., 1995), indicative of HPA hyperactivity, and mice with low or absent β-E have enlarged adrenal glands, suggesting chronic upregulation of the HPA axis with decreased β-E levels (Grisel et al., 2008).

The present study, along with earlier studies in our lab, provides evidence of a moderating effect of β-E on the behavioral response to stress (Grisel et al., 2008; Barfield et al., 2010), implicating a role for this peptide in coping behavior. In particular, we found an effect of interaction between genetic predisposition and environmental stressors on anxious behavior in mice. Behavioral differences between “genetically vulnerable” (low or absent β-E) and “genetically resistant” mice emerged when mice were exposed to a stressor before the NSF test. These data suggest that low β-E levels impair the ability to return to a basal state following stress exposure, and thus compromise coping ability. Considering the evidence for heritability of stress-induced HPA axis activity together with the findings presented here, it is possible that genetically determined differences in sensitivity of the β-E system to stress contribute, at least in part, to heritable differences in vulnerability to developing anxiety and depression (Charmandari et al., 2005; Hegadoren et al., 2009; Merenlender-Wagner et al., 2009).

MDD and anxiety disorders affect a significant portion of the nation, with a lifetime prevalence of ~20% for MDD and 28% for anxiety disorders (Kessler et al., 2005). Although the neural mechanisms involved are poorly understood, evidence from clinical and pre-clinical studies implicates the role of HPA axis abnormalities in the pathophysiology of mood and anxiety disorders (Carroll et al., 2007; see Arborelius et al., 1999, for review). Thus, genetically mediated interindividual differences in HPA axis activity may help explain why some individuals are particularly vulnerable, and others resilient, to anxiety and depression (Holsboer et al., 1995; Wüst et al., 2000; McEwen, 2002; Vreeburg et al., 2010). Altogether, our findings suggest that β-E facilitates coping behavior. Low levels of this peptide may impair the coordination and dynamics of the stress response, thereby enhancing vulnerability to stress-related psychopathology. Further investigation of the role of β-E in allostasis of the stress response may yield insight into the etiology of anxiety and depression.

### Conflict of interest statement

The authors declare that the research was conducted in the absence of any commercial or financial relationships that could be construed as a potential conflict of interest.
